# Couples infected with hepatitis viruses and assisted reproductive technologies: management and outcome

**DOI:** 10.1186/1471-2334-13-S1-P110

**Published:** 2013-12-16

**Authors:** Andreea Velişcu, Bogdan Marinescu, Mihai Mitran, Marilena Băluță, Lucia Costoiu, Cristina Damian

**Affiliations:** 1 Clinical Hospital of Obstetrics and Gynecology “Prof. Dr. Panait Sârbu”, Bucharest, Romania

## Background

How do infected couples (one or both partners) with hepatitis viruses and infertility achieve pregnancies? We need to assess the quality of embryos, sperm and pregnancies rates.

## Methods

We performed a retrospective study in our clinic over a period of 5 years. We selected all couples infected with hepatitis viruses (one or both partners) that underwent an in vitro fertilization procedure or intrauterine insemination. We studied pregnancy rates, birth rates compared to a control group and quality of embryos and sperm (in infected males).

## Results

The sperm parameters are lower in infected men when compared to the control group and there are fewer embryos for the infected couples, but pregnancies rates are comparable between the two groups.

## Conclusion

All infected couples should have access to reproductive techniques with normal pregnancy rates.

